# The Effect of Conditional Probability of Chord Progression on Brain Response: An MEG Study

**DOI:** 10.1371/journal.pone.0017337

**Published:** 2011-02-25

**Authors:** Seung-Goo Kim, June Sic Kim, Chun Kee Chung

**Affiliations:** 1 Interdisciplinary Program in Cognitive Science, Seoul National University, Seoul, Korea; 2 MEG Center, Department of Neurosurgery, Seoul National University College of Medicine, Seoul, Korea; Cuban Neuroscience Center, Cuba

## Abstract

**Background:**

Recent electrophysiological and neuroimaging studies have explored how and where musical syntax in Western music is processed in the human brain. An inappropriate chord progression elicits an event-related potential (ERP) component called an early right anterior negativity (ERAN) or simply an early anterior negativity (EAN) in an early stage of processing the musical syntax. Though the possible underlying mechanism of the EAN is assumed to be probabilistic learning, the effect of the probability of chord progressions on the EAN response has not been previously explored explicitly.

**Methodology/Principal Findings:**

In the present study, the empirical conditional probabilities in a Western music corpus were employed as an approximation of the frequencies in previous exposure of participants. Three types of chord progression were presented to musicians and non-musicians in order to examine the correlation between the probability of chord progression and the neuromagnetic response using magnetoencephalography (MEG). Chord progressions were found to elicit early responses in a negatively correlating fashion with the conditional probability. Observed EANm (as a magnetic counterpart of the EAN component) responses were consistent with the previously reported EAN responses in terms of latency and location. The effect of conditional probability interacted with the effect of musical training. In addition, the neural response also correlated with the behavioral measures in the non-musicians.

**Conclusions/Significance:**

Our study is the first to reveal the correlation between the probability of chord progression and the corresponding neuromagnetic response. The current results suggest that the physiological response is a reflection of the probabilistic representations of the musical syntax. Moreover, the results indicate that the probabilistic representation is related to the musical training as well as the sensitivity of an individual.

## Introduction

### Harmonic progression and probabilistic learning

**Figure 1:**
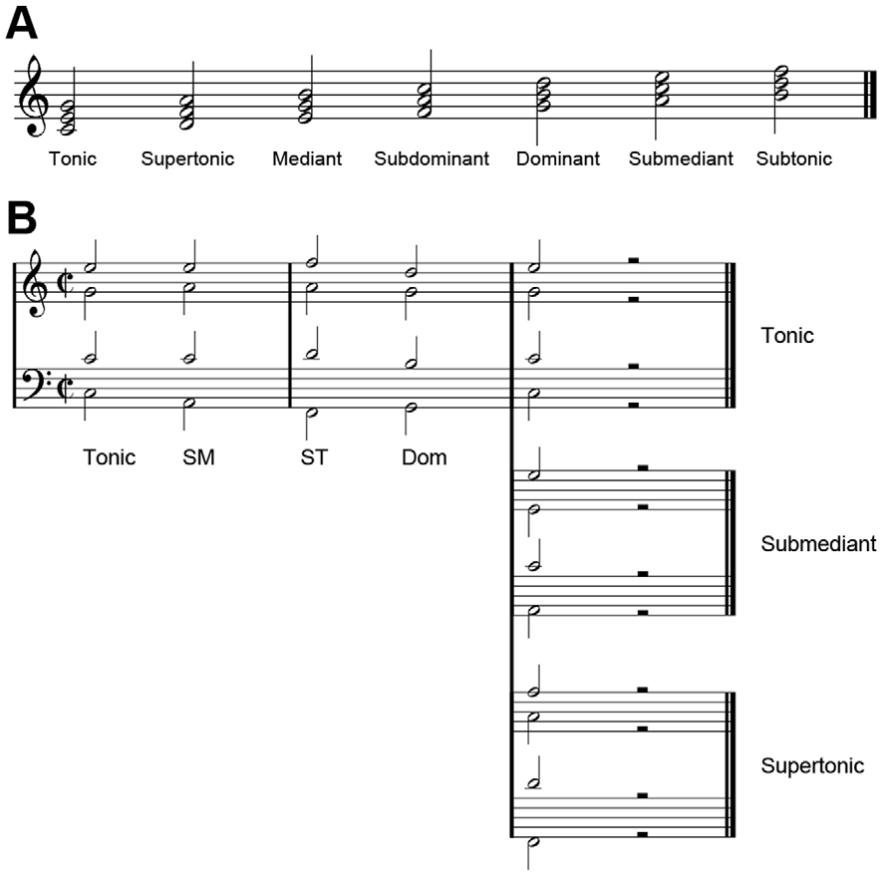
Illustrations of chord functions and sequences used. **A.** Chord functions in C major. **B.** Chord function sequences used in the present experiment. Conditions differ according to the ending chord function: tonic, submediant (SM) or supertonic (ST). Dom, Dominant.

Recent electrophysiological and neuroimaging studies have explored how the musical syntax, particularly that of harmonic progression, in Western music is processed and which regions of the human brain are involved [1]. Koelsch and colleagues [2] have reported that the violation of the harmonic expectancy elicits a specific event-related potential (ERP) component called an early right anterior negativity (ERAN). An ERAN is a negative component that peaks between 150–210 msec after the irregular chord onset and that occurs predominantly in the right frontal region. An irregular chord, or ‘chord function’ in relation to the current key (**Figure 1A**), which evokes such a negativity can include notes out of the current key (such as Neapolitan sixth [2] or double dominant [3]) or only in-key notes (such as supertonic [3]). Modulating factors of the ERAN have been extensively investigated. The latency and amplitude of the ERAN differ by attention [4], prior short-term exposure [5], ages [1], and musical training [6], [7]. Especially for the effect of gender, in female participants, the early anterior negativity did not show right predominance but bilateral scalp distribution [8]. For this reason, in other studies [4], [9], [10], the negative ERP component elicited by irregular chords has been simply termed as early anterior negativity (EAN).

The possible underlying mechanism of the ERAN may be assumed to be probabilistic learning. As suggested in [1], the detection of the irregularity of chord progression requires the perception of simultaneously occurring tones as a chord function in the tonal hierarchy [11] and the knowledge about the norms of chord function changes. With repeated experience with Western tonal music, a listener would develop a probabilistic representation of the chord progressions which is essential to form an expectation for further chord functions and also to elicit an ERAN response when the expectation is violated [1]. Whereas 4-month-old infants do not show ERAN responses to irregular chord transitions, 2.5-year-old children do, though they are smaller than those seen in adults [1]. The behavioral evidence of the ability to use the probabilistic properties of the tone sequence in 8-month-old infants as much as adults supports that the typical development of the mechanism for statistical learning takes place quite early in the normal population [12]. The mean latency of ERAN responses in 5-year-old normal children (230–240 ms) was longer than that in adults [13] and the ERAN components peak around 320 ms after the onset in both 5-year-old and 9-year-old normal children [14]. At the age of 11, the ERAN latency is similar to that of adults (around 180 ms) [15] regardless of musical training. These lines of evidence suggest that the underlying neural mechanism of the ERAN response is developed by the accumulated exposure to music through the early years of life.

### Rapid learning in short-term exposure

While the ERAN response is assumed to be dependent on repetitive exposure over a long time and following long-term memory, a violation within a simple sound pattern after repetitions in a short period of time is known to elicit a typical negative ERP response with a latency of 150–210 ms after the onset of the deviant occurrence. This response is termed mismatch negativity (MMN) [16], [17]. The MMN response is interpreted as a brain signature of the detection of an aberration in the represented pattern within the ‘on-line’ auditory environment involving short-term memory [17]. It is noteworthy that the deviant stimulus or the ‘oddball’ that elicits the MMN is defined by the rarity of its occurrence during the ongoing sound presentation and not the properties of the sound itself such as its frequency, duration or intensity. It is well shown in the ‘roving paradigm’ in which a certain stimulus switches its role from a standard stimulus to a deviant stimulus by its relative frequency within a narrow sliding time-window and vice versa [18]. The probability of the deviant stimulus [19] and the number of repetitions of the preceding standard stimulus [18], [20] modulate the amplitude of the MMN.

In the domain of music, Loui and colleagues [10] demonstrated that the probabilistic representation of novel pitch sets in an artificial musical scale can be learned and facilitated by an hour of exposure as they found that the EAN responses were elicited by the rare (20% of presentation) pitch sets. Note that the presentation of the deviant pitch sets with equiprobability to the standard pitch sets prior to the main experiment resulted in indistinguishable ERP patterns. EAN responses were elicited only when the deviant pitch sets were presented with a smaller probability than the standard pitch sets and the amplitude of the EAN increased as the exposure accumulated during the 1-hour-long experiment. These support the notion that the neural mechanism underlying the EAN response is related to a rapid ability to learn probabilistic patterns of the occurrences [21].

### Aims and hypotheses

As in the previous literature, the modulation of probability of the presentation of a single tone or simultaneous tones on the neural response has been well explored, however the role of the probability of the sequential structure of music in terms of harmony remains to be investigated. Some previous experiments addressed the effect of the degree of expectancy while investigating the effect of harmonic expectancy violation in actual musical pieces [22], [23]. However, various kinds of irregular chord progressions were mixed within a single level (i.e., ‘unexpected’) of expectancy manipulation among other levels (i.e., ‘expected’ or ‘very unexpected’) in those works and so the precise effect of probability has not been revealed to date.

The aim of the present study is to provide a direct examination of the effect of probability of chord progression on brain response using magnetoencephalography (MEG). If an EANm (as a magnetic counterpart of the EAN component) response is based on a probabilistic representation built up by experience, the amplitude of the EANm response to a certain chord progression will correlate with the corresponding probability. Also, if the EANm response is based on a probabilistic representation that is facilitated by repetitive and intensive exposure, this relationship will be likely to be enhanced by prior musical training. Finally, if there are individual differences in the probabilistic representation on the sequential structures in music, it will be reflected in the neural response related to the rarity of musical events and also will correlate with the individual ability to discriminate them.

## Materials and Methods

### Conditional probability from J. S. Bach's Chorale

The probability of chord progressions in the present study is defined as the conditional probability of a bigram regarding a single preceding chord function. The conditional probability can be computed as
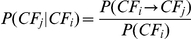
where 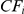 is a certain chord function ***i,*** 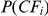 is the probability of the occurrence of the chord function ***i***, or an unigram, and 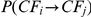 is the probability of the occurrence of a chord transition from chord function ***i*** to chord function ***j***, or a bigram [24]. Since the total number of possible bigrams in a certain corpus is invariant, the conditional probability can be obtained by simply dividing the number of corresponding occurrences. This approach analyzing the chord transition as in bigrams may be limited for the chord progression in practice where constructing chord transitions may get involved in complex contexts and long-term chord dependencies [25]. For simplicity, however, the bivariate relation is an initial approach for analyzing the sequential structures in music such as in recent computational musicological literature [21], [25], [26].

Though the empirical probabilities of chord transitions within the entire experience of an individual are most likely to be related to the probabilistic representation of the individual, calculating such probabilities is an unrealistic job since we do not keep track of all playlists of an individual over their lifetime. Instead, a computational corpus study on J. S. Bach's Chorale [24] is employed in order to approximate the conditional probabilities of chord progressions. Note that it is not necessarily thought that the individual musical experience should follow the empirical probabilities in J. S. Bach's Chorales. Though the chord transition matrices would differ along with various musical corpora [21], J. S. Bach's chorales have been considered to be important references for the development of musical tonality, which is central to Western music theory, even for today [24]. Thus it is possible to assume that the empirical probabilities in Bach's chorales are approximations of those in the whole Western tonal music.

For the experimental feasibility, while the preceding musical context was controlled as invariant, three ending chord functions were selected: tonic (I; conventional notation for chord functions in Roman numerals), submediant (vi) and supertonic (ii) (**Figure 1B**). Since the 4th chord functions in all sequences were constantly dominant (V), the corresponding conditional probabilities of the conditions are computed using the reported absolute frequencies [25] as: P(I|V) = 1042/1386 = 0.7518, P(vi|V) = 147/1386 = 0.1061 and P(ii|V) = 60/1386 = 0.0433. Supertonic has been previously used as an irregular ending chord for eliciting the ERAN responses reliably [3], [5], [13]. Submediant is one of the chord functions used in the ‘unexpected’ condition in previous literature [22]. Note that the conditional probability of the transition from dominant to submediant is greater than that of any other case except for that of the transition from dominant to tonic. This transition is regarded as one of the important examples of the suspended resolution in music theory [27] though there are other practical issues for effective usage. Thus submediant is assumed to provide the intermediate level of rarity between tonic and supertonic to the listeners in the present study.

### Materials

The stimuli of the present experiment were chord sequences consisting of five chords in four-parts which are similar to those of previous literature [2], [28], [29]. Chord sequences were composed accordingly to the classical conventions of harmony progressions including voice-leading and the avoidance of consecutive or hidden perfect fifths and eighths (**Figure 1B**). The first four chord functions of the sequence in all cases were tonic, submediant, inverted supertonic, and dominant, in that order. The ending chord function was tonic, submediant or supertonic. This variation determined the stimulus condition.

The sequences were transposed to include all of the possible 12 major keys and were played at 100 BPM (600 ms for each chord; 3,600 ms for each sequence) using *Cubase 5* (Steinberg, Hamburg, Germany) with a VST instrument, *The Grand 3* (Steinberg, Hamburg, Germany) for a piano timbre (*Bösendorfer 290 Imperial grand*). Then the intensity of the exported wave file (sampling rate: 44.1KHz; 16-bit; stereo; Windows PCM) was normalized using *Cool Edit Pro 2.1* (Syntrillium Software Corporation, Phoenix, AZ, USA).

Note that all ending chord functions were already played within the sequence before the 5th position (i.e., in the tonic condition, tonic occurred in the first and the fifth position; in the submediant condition, submediant occurred in the second and fifth position; and in the supertonic condition, supertonic occurred in the third and fifth position) in order to rule out that the responses were due to acoustic deviants in echoic memory [3].

### Participants

12 healthy non-musicians (mean age: 24.6±2.6 years; age range: 21.5–29.3 years) and 10 musicians (mean age: 23.9±3.5 years; age range: 20.4–31.6 years) participated in the experiment at the MEG center, Seoul National University Hospital (Seoul, Korea). All participants were right-handed (mean Edinburgh Handedness coefficient [30]: 92.6±9.2 for non-musicians, 88.6±12.6 for musicians). Musicians had studied musical instruments in music colleges and had received training for at least 15 years since the age of 5. Nine musicians had practiced piano and one musician had practiced violin as their primary musical instruments. Besides, musicians had also trained for violin, cello, flute or piano as their secondary instruments for more than a year. Many (8 out of 12) non-musicians had experienced musical lessons, mostly for piano, as an extracurricular activity for about 3 years but no non-musicians had been practicing any musical instrument even occasionally at the time of the experiment except one participant. Even if we included the non-musician who had been actively practicing, it would not change the significance of the statistical results in general. However, the subject was excluded from the further analysis to prevent any possible confounding. All musicians had knowledge about the theory of harmony whereas 3 non-musicians reported that they had a basic understanding. Participants were recruited as volunteers from Seoul National University (Seoul, Korea) and Yonsei University (Seoul, Korea) communities. All participants gave written informed consent before the experimental sessions and were paid 10,000 KRWs (about 9 USDs) per hour for their participation. The materials and research protocols were approved by the Institutional Review Board of Seoul National University Hospital (H-1001-020-306).

### Procedure

The experiment consisted of two sessions: a MEG recording session and a behavioral test session. For the MEG recording session, participants were seated in a magnetically shielded room with their back supported by a chair, their arms on a table, and their legs on leg rests. The chord sequences were presented to the participants at approximately 65 dB Sound Pressure Level for the maximal amplitude using the *STIM2* system (Neuroscan, Charlotte, NC, USA) via MEG-compatible tubal insert phones (*Tip-300*, Nicolet, Madison, WI, USA).

A block consisted of 100 sequences. Each sequence ending with either tonic, submediant or supertonic was presented 30 times per block in a pseudo-random order. In addition, 10 ‘staccato’ sequences were also presented. In the ‘staccato’ sequences, a single chord either in the second, third, forth or fifth position was played 1/16 the duration of the other chords (i.e., ‘staccato’: 37.5 ms, others: 600 ms) with a following quick decay (75 msec). This is analogous to the ‘fadeout’ chords previously used in [4], [10]. The participants' task was to detect the staccato sequences and respond by pressing a button with their right index finger. This task was designed to maintain the arousal level of the participant and preserve their attention to the auditory stimuli. The neuromagnetic responses to ‘staccato’ sequences were not included in further analysis but the behavioral performances were used to check the participants' attention level. No participants made more than two incorrect responses including false alarms and misses for the whole trials (error rates <0.33%). Six blocks were administrated with breaks through the MEG session which resulted in 180 sequences presented for each condition in total.

After the MEG recording session, a behavioral test session was conducted. Participants were instructed to determine the ending chord function and respond using a keypad and sufficiently practiced the task to understand it. The key assignment was made as follows: button #1 for tonic, button #2 for submediant and button #3 for supertonic. All three conditions (tonic, submediant and supertonic) were presented in the 12 major keys in a pseudo-random order resulting 36 sequences in a block. Using the same stimuli and apparatus used in the MEG recording, three blocks were administrated with breaks. Experimental sessions consisting of preparation, MEG recording and behavioral testing took approximately 2 hours in total.

### MEG recording

Magnetic signals from the cerebral cortices were recorded using a 306 channel *Elekta Neuromag system* (Elekta Neuromag Oy, Helsinki, Finland). The MEG system has 102 identical triple sensor elements (one magnetometer and two gradiometers which are oriented perpendicularly to each other) in a helmet-shaped array. The sensors provide independent measures of magnetic field strength due to their orthogonal disposition. MEG signals were recorded using a 0.1–200 Hz band pass filter at a sampling rate of 600.615 Hz. Vertical and horizontal electrooculograms (EOG) and electrocardiograms (ECG) were obtained simultaneously with the MEG recording in order to remove ocular and cardiac noise later. Four Head Position Indicator (HPI) coils were attached to the participant's scalp to monitor the head position within the sensory array. The locations of HPI coils with respect to three anatomical landmarks (the nasion and the bilateral preauricular points) and additional points on the scalp were identified using a 3D digitizer (*Fastrak*, Polhemus, Burlington, VT, USA). Prior to the MEG recording, an experimenter checked that the head was positioned correctly in the helmet and instructed participants not to move throughout the entire block of MEG recording. The information obtained on head position was used to compute the origin and radius sphere model in source analysis.

### MEG data analysis

*MaxFilter* 2.1.13 (Elekta Neuromag Oy, Helsinki, Finland) was applied to raw MEG signals in order to eliminate environmental magnetic noise, detect the ‘bad channels’ or those having exceedingly large amounts of noise and align the MEG data across blocks within the subject. Then the MEG signals were averaged using *MATLAB 7.5.0.342* (MathWorks Inc., Natick, MA, USA) with a *MATLAB* toolbox (*Fiff Acess 1.2*, Brain Research Unit, Low Temperature Laboratory, Helsinki University of Technology, Helsinki, Finland) and in-house codes. Epochs were defined as data from 200 ms before and 500 ms after the target chord onset. Epochs having EOG artifacts were discarded by a threshold defined though visual inspections. Epochs free of artifacts numbered more than 140 epochs in all cases (mean: 160.6±14.3 epochs) and were averaged for each of three conditions. Whenever an excessive number of epochs were rejected due to EOG artifacts (rejection rates >45%) as was in case for 1 non-musician and 1 musician, a sufficient number of epochs could not be collected in order to achieve the competent level of signal-to-noise ratio (SNR) as were collected for other participants. Thus, the data from these 2 participants were excluded from further analyses.

Source analysis of MEG data was performed using the spatio-temporal source analysis tool in *BESA 5.1.4.40* (MEGIS Software GmbH, Gräfelfing, Germany) [31]. A band-pass filter (1–20 Hz, zero phase) was applied to the averaged MEG signal. A homogeneous spherical model was used of which the origin and radius were determined based on the anatomical landmarks digitized before the MEG recording. Multiple equivalent current dipoles (ECDs) were fit according to the following criteria. First, to account for the primary neural activities to the auditory stimuli, the generators of P2m (peaks with the latency of 180–190 ms within the sensors over temporal lobes) [32] were localized in the average of all in-key chords using the Genetic Algorithm as an iterative multiple ECDs fitting method with a symmetry constraint on the position of ECDs [33]. All in-key chords at all positions (>1,000 epochs) were pooled to acquire higher SNR in a similar way as in [29]. The resulting ECDs were fit on the bilateral auditory cortices, presumably the Heschl's gyri [34]. Next, the additional two bilateral ECDs for EANm responses were fit on the frontal regions within the time-window of 140–220 ms after onset while the former temporal ECDs were fixed with respect to locations and orientations. This additional ECD fitting was performed on the MEG data from the supertonic condition because frontal activity in response to supertonics is observed consistently in the literature [3], [5], [13]. The frontal ECDs were localized on the bilateral inferior frontal gyri (IFG). A multiple ECD model of which the goodness of fitting (GOF) or the ratio of explained variance exceeded 80% was considered as acceptable. Finally, the activities of four ECDs were estimated for each condition (tonic, submediant and supertonic) by the signal-space projection method [35] implemented in *BESA*. The set of four multiple ECDs served as a spatio-temporal filter for the MEG signals. The estimated activities of ECDs were exported for further statistical analysis.

### Statistical analysis

The maximal absolute values of ECDs activities of the bilateral IFGs within the time-window of 100–170 ms and 180–250 ms after onset were obtained as the activities peaked at around 135 ms and 220 ms after onset. All analyses were done in both time-windows. The difference in amplitude between the right and left IFG activities in the present data was not significant (*F* (1,113) = 0.47, *p* = 0.4963 for 100–170 ms; *F* (1,113) = 0.08, *p* = 0.7751 for 180–250 ms) as some participants showed no lateralized EAN/ERAN responses in the previous literature [4], [8], [9], [10]. Thus the bilateral IFG activities were then averaged. The effects of probability and musical training in EANm response were tested by repeated measures ANOVA using the *SPSS 12.0.1* software package (SPSS Inc., Chicago, IL, USA).

Because auditory responses typically vary to a great degree across individuals, it is possible that the effect of conditions may be smaller than the variance across individuals. To minimize the inter-individual variability when applicable, the amplitudes of the ECD activities were normalized by dividing the magnitude of ECD of the tonic condition for each individual as in other MEG studies [34], [36].

Additionally, for better explanation of the data, a General Linear Model (GLM) using a second-order model was applied to test and estimate the effect of musical training and the interaction with the conditional probability on EANm responses. The GLM is a flexible and general statistical framework encompassing a wide variety of fixed effect models [37]. For the purpose of the test, a GLM was considered as the following:
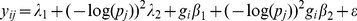
where 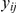 is the normalized EANm response of the individual subject ***i*** to the chord function ***j***, 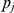 is the conditional probability of the chord function ***j*** (0.7518, 0.1061 and 0.0433), 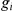 is the group index of the individual ***i*** (non-musician = 0 and musician = 1) and 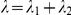 and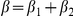 are the unknown parameters to estimate. 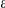 is assumed to be zero mean Gaussian noise. The null hypothesis (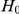) that assumes the main effect and the interaction effect of the musical training to be zero and the alternative hypothesis (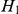) that assumes them to be non-zero are as
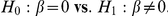



## Results

### Source analysis

**Figure 2:**
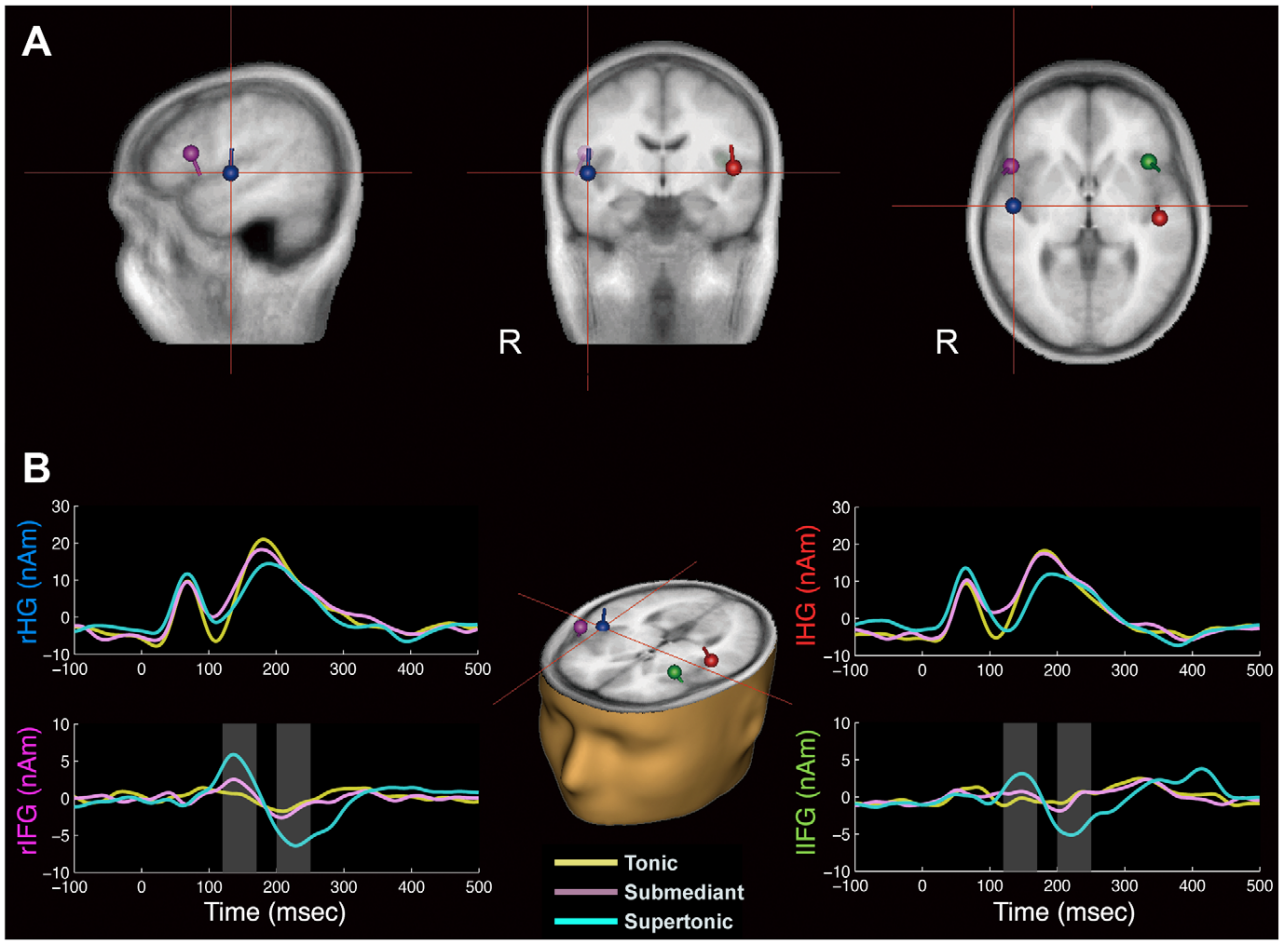
Locations and activities of multiple ECDs. **A.** Multiple ECDs of an individual superimposed on a sagittal (left panel), a coronal (middle panel) and an axial (right panel) plane of stereotactic template MRI orientated in a radiological convention (left to right). ECDs are located on the left Heschl's gyrus (red), the right Heschl's gyrus (blue), the left inferior frontal gyrus (green) and the right inferior frontal gyrus (magenta). **B.** Averaged ECD activities across all participants (n = 19) for tonic (yellow), submediant (purple) and supertonic (cyan) conditions. The shaded areas indicate the time-windows used in statistical analysis.

Multiple ECDs were localized bilaterally approximately on the Heschl's gyri (HG) and the inferior frontal gyri (IFG) (**Figure 2A**). The means and standard errors of means of the locations of ECDs in the head coordinate system are listed in **Table 1**.

**Table 1:** Means and standard errors of the means (in parentheses) of the locations of multiple ECDs in the head coordinate system (n = 19).

Mean (SEM)	x (mm)	y (mm)	z (mm)
lHG	−42.50 (1.22)	−0.73 (1.64)	46.73 (1.31)
rHG	46.48 (1.30)	6.12 (1.98)	47.90 (1.34)
lIFG	−36.34 (2.26)	16.83 (5.06)	67.15 (3.42)
rIFG	42.20 (2.54)	18.67 (5.00)	64.89 (3.46)

lHG, left Heschl's gyrus; rHG, right Heschl's gyrus; lIFG, left inferior frontal gyrus; rIFG, right inferior frontal gyrus.

The average location of IFG dipoles was anterior (15.06 mm) and superior (18.70 mm) to that of HG dipoles. Hotelling's ***t^2^*** tests for the 3D coordinates between of the HG dipole and the IFG dipole in each hemisphere verified that the locations of the ECDs are clearly distinguishable (***t^2^*** **_left_** (3, 34) = 38.81, *p*<0.0001; ***t^2^*** **_right_** (3, 36) = 22.41, *p* = 0.0008).

### Effects of conditional probability and musical training

The estimated activities of IFG dipoles showed a deflection around 135 ms and 220 ms after onset during the conditions of submediant and supertonic compared to the tonic condition (**Figure 2B**). The EANm response within the former time-window [100,170] is referred as EANm_1_ and the one within the latter time-window [180,250] is referred as EANm_2_. Repeated measures ANOVAs on unnormalized EANm responses were performed to determine the effects and the interaction of the chord types (within-subject factor) and the musical training (between-subject factor). For EANm_1,_ the test for sphericity was significant (***x*** ^2^ (2) = 10.093, *p* = 0.006) using Mauchly's criterion (W = 0.532), i.e., the assumption for sphericity was invalid [38]. As the Greenhouse-Geisser's epsilon was smaller than 0.75, the Greenhouse-Geisser's correction was adapted as suggested in [39]. The effect of chord functions (*F* (1.363, 23.163) = 17.546, *p*<0.0001) was significant but the interaction with musical training (*F* (1.363, 21.861) = 2.513, *p* = 0.118) was not significant. For EANm_2_, the sphericity test was not significant (***x*** ^2^ (2) = 3.312, *p* = 0.191) thus sphericity assumption was used. The effect of chord functions (*F* (2,34) = 46.278, *p*<0.0001) and the interaction with musical training (*F* (2,34) = 6.406, *p* = 0.004) were both significant.

**Figure 3:**
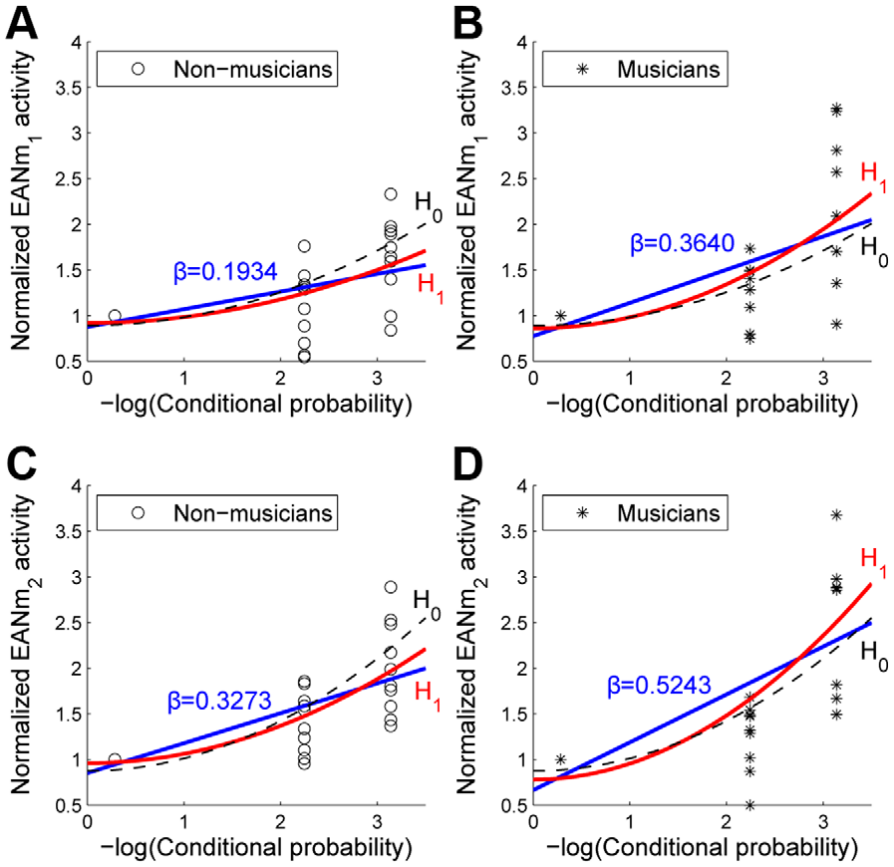
The relation between negative logarithmic conditional probabilities and normalized EANm activities. Linear fitting lines (blue) and quadratic fitting curves for a null hypothesis (black dashed curves) and for an alternative hypothesis (red curves) were superimposed on scatter plots for the non-musicians (**A**) and the musicians (**B**) for the time-window of 100–170 ms as EANm_1_ and for the non-musicians (**C**) and the musicians (**D**) for the time-window of 180–250 ms as EANm_2_. Each data point on the scatter plot represents an individual normalized EANm activity for each condition. The slopes of linear fitting lines (*β*) are noted.

The performed ANOVAs assuming the chord functions as a categorical variable could only indicate the mean difference amongst levels but not the amount of the effect of the conditional probability of the chord function on EANm response. Linear regressions on the EANm response with the conditional probabilities as a regressor were estimated in each group (Figure 3). The scale for the conditional probability was logarithmic since the frequency of chord transitions follows Zipf's distribution [24]. In all cases, the null hypothesis that assumes the effects of regressors to be zero was rejected (*p*'s<0.0027). That is, in both cases, the EANm responses significantly correlated with the conditional probability of chord functions. The slope (*β*) of the linear regression was higher in the musicians group than in the non-musicians (*β*_non-musicians_ = 0.3640>*β*_musicians_ = 0.1934 for EANm_1_; *β*_non-musicians_ = 0.5243>*β*_musicians_ = 0.3273 for EANm_2_).

Additionally, to test precisely the main effect and the interaction effect of musical training, a GLM was performed (see *2. 7. Statistical Analysis*). The square sums of errors of the null and alternative second-order models were compared. The model considering the effects of musical training fit significantly better (*F* (2,53) = 5.8343, *p* = 0.0051 for EANm_1_; *F* (2,53) = 5.8439, *p* = 0.0051 for EANm_2_). The linear fitting lines (blue) and quadratic fitting curves (red) for each group are shown on scatter plots in **Figure 3** in order to indicate the effect of musical training.

### Individual differences in behavioral test

**Figure 4:**
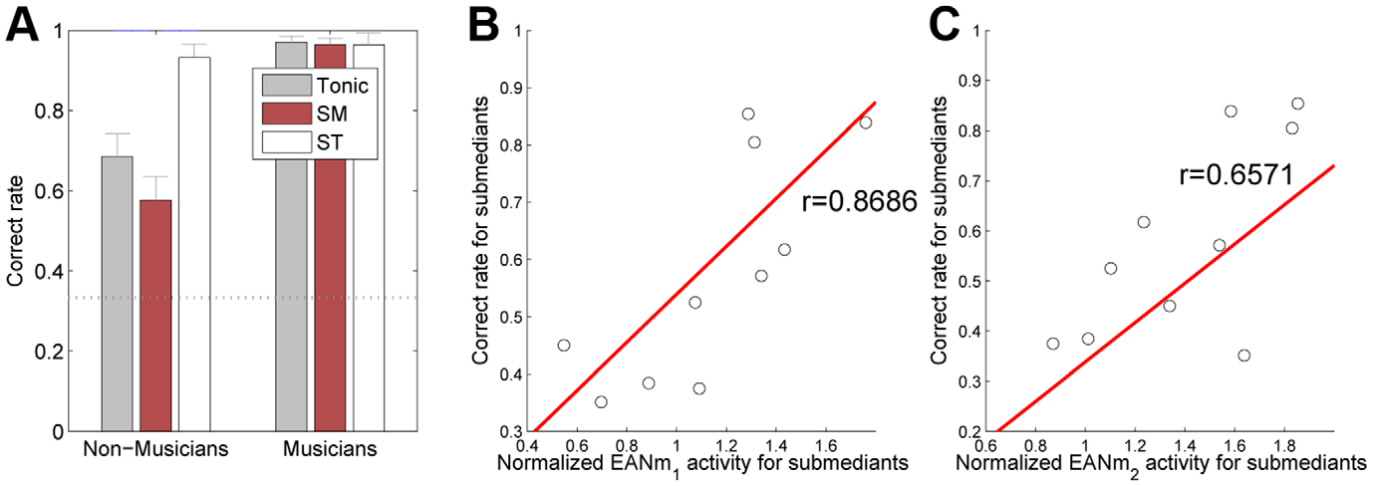
Mean correct rates on the behavioral test and correlation with normalized EANm activities. **A.** Mean correct rates by types (tonic, submediant, supertonic) and groups. Error bars indicate the standard errors of the means. The dotted horizontal line indicates the chance level (1/3). **B.** Scatter plot of normalized EANm activities vs. correct rates for submediants in non-musicians for the time-window of 100–170 ms as EANm_1_. A linear regression line is indicated (red) with the Pearson correlation coefficient (***r***) noted. **C.** Scatter plot for the time-window of 180–250 ms as EANm_2_.

Performances on the behavioral tests in which the participants were asked to identify the ending chord function are shown **Figure 4A**. The distributions of the correct rates of the musicians were highly skewed to the maximal values as the most of (7 out of 9) of the participants with musical training made correct answers over 0.95 regardless of the type of stimulus. Thus no correlation analyses were performed with the behavioral data of the musicians. On the other hand, for the non-musicians, the mean correct rates ranged from 0.49 to 0.94. The correlation between the correct rates for the submediant conditions and normalized EANm activities was significantly positive (*r* = 0.8686, *p* = 0.0011 for EANm_1_ in **Figure 4B**; *r* = 0.6571, *p* = 0.0390 for EANm_2_ in **Figure 4C**) while the correct rates in other cases did not significantly correlate.

## Discussion

In the present data, the EANm responses were elicited by rare chord progressions, which is consistent with previous studies [3], [13], [22]. Since the rare chord functions were already played within the sequence before the position of the ending chord, the possibility of the neural responses being due to the sensory deviance can be discarded. Rather, the response is likely to be due to the relationship between the preceding musical context and the following chord function and to be related with the cognitive components in the music-syntactic processing [40].

The ECDs for frontal activities were localized to bilateral IFGs in accordance with the previous MEG and fMRI studies [28], [29]. The role of Broca's area and its right homologue including the IFG in the processing of the syntactic structures has been suggested to be critical and shared in both music and language [41]. Previously, activities produced while processing the syntactic violation in spoken language were also localized to the IFGs by using MEG [42], similarly to that seen in the present study. In addition, in a study using fMRI and EEG, a neural generator on the right IFG, along with some on the bilateral superior temporal gyri, was found to involve in producing MMNs [41]. As discussed in [43], the involvement of the opercular part of the IFGs is suggested to be related to auditory memory and attention. Moreover, higher cognitive processes can be related to the functional role of IFG [10] because the frontal activity to deviants was not directly related to the physical differences in stimuli but the perceived differences as in [44]. A recent EEG study also reported the abnormal scalp potential distribution in musical syntax processing in the patients with lesions in the left IFG, or Broca's area [45]. More intriguingly, a single case in this study showed an impaired discrimination of chord functions and ERAN response in a patient who was in the early stage after injury that may implicate any recovery or compensation was yet to be done. It demonstrates the deep involvement of the left IFG in musical syntax processing.

The relationship between the negative logarithmic conditional probability and the relative magnitude of the EANm response was significantly positive, suggesting that, the rarer the occurrence, the greater the neural response elicited in an exponential fashion. The general relationship was explored in former studies comparing binary conditions [2]. But for the first time, the present study explains the neural responses as a function of the probability. The observed EANm responses were fit in terms of the empirical probability of the Western corpus. This suggests that the probabilistic representations in the listeners might follow the canonical instances of Western musical syntax in general.

Furthermore, for monophonic music, or melodies, the conversion of computational, behavioral and electrophysiological evidences has been recently reported [46]. In that study, the ratings of expectedness of the participants highly correlated with the estimation of a model based on the probabilities [47] and the ERP and neural oscillatory activities were clearly distinguished by the probabilities as well. The negative correlation between the conditional probability and neuromagnetic response shown in our study also supports the ability of the brain to learn statistical regularities in its perceptual input and to use these regularities to predict future events [46].

The effect of music training was significant on the EANm response interacting with the effect of conditional probability. The responses in musicians to the rare occurrence of the chord progression were enhanced in the present data as were seen in previous MMN studies [48], [49] and ERAN studies [6], [50]. The musicians in the present study had training in performing the Western classical music and the currently used musical corpus is actually considered as a milestone of the Western classical music [24]. It may be possible to conjecture that the musicians have a more congruent probabilistic representation to the corpus in the present study as they are more likely to have a greater amount of exposure to the music of a similar style compared to the non-musicians.

The individual differences in behavioral sensitivity to the chord progressions were associated with the neuromagnetic responses. The performances in the other conditions than the submediant conditions were not normally distributed, possibly for the low degree of difficulties. It was not possible to see the individual differences in those biased measures. However, the sub-correct rates for submediants most highly correlated with the overall correct rates in the non-musicians (*r* = 0.9635, *p*<0.0001) which means that the majority of errors occurred in the submediant condition. In accordance with the previously reported association between the physiological responses and the behavioral measures [10], the EANm herein may reflect the individual competence at identifying subtle aberrations. However, it is noteworthy that there seem to be more complex mechanisms involved in between the early neural response and the behavioral discrimination. Peretz et al. reported that even in amusia brains, ill-tuned notes can occur early negativities, dissociated with their abilities to judge incongruities [51]. But the error detection and awareness are not irrelevant either since out-of-key (but well-tuned) notes made only small effects in amusia brains unlike the normal brain.

A further implication of the relationship investigated in the present study can be associated with the sensitivity of the ventral prefrontal cortex, including the IFG, to the probability of stimulus. In a previous fMRI study using go/no-go paradigm, when a specific no-go stimulus (always "X") was repeated frequently the activation of the ventral prefrontal cortex decreased and vice versa [52]. Furthermore, the response to the syntactic violation in language, known as early left anterior negativity (ELAN [53]), was found to be modulated by the manipulation of the probability [54]. In that EEG study, an illicit sequence elicited a greater ELAN response when the probability of the target clause was low. The current finding of the relationship between probability and EANm response suggests a sensitive representation of probability of chord progression and extends the previous findings of the cortical sensitivity to probability to the musical syntax domain. Moreover, the correlation found in the present study between individual neural responses and behavioral performance supports the involvement of the ventral prefrontal regions in the individual sensitivity to the probability of chord transitions [52].

For additional issues, we found a slight difference in peak latencies between hemispheres in the time-window of 100–170 ms after onset for supertonics in the IFG ECDs (Left IFG = 155.84±17.09 ms; Right IFG = 148.34±22.26 ms; *t* (18) = 2.0395, *p* = 0.0564) but the difference was not significant in each separate group (*t* (9) = 1.2765, *p* = 0.2337 for non-musicians; *t* (8) = 1.5407, *p* = 0.1620 for musicians). Thus we assume that it is likely to be a false positive.

We have also observed a trend of a late deflection (around 400 ms) evoked by supertonics in the left IFG ECD in the musicians (**Figure 2B**). This may be reminiscent of the P3-like responses which only musicians elicited to the ‘very unexpected’ chord in the previous literature [22]. However, the maximal amplitudes between the time-window of 380–420 ms in the musicians were not significantly different across the conditional probability (*F* (2,26) = 0.82, *p* = 0.4539).

There are some limitations in the present study to mention. The first limitation is that the adopted probability in the current study could be different from ones in the individual probabilistic representations. The conditional probabilities of chord transitions in corpora differ by specific musical styles (e.g., Baroque or Western popular music) [21] and personal experiences of music may be quite different. Even accounting for the individual differences in the ability of pattern extraction from the auditory stimuli [55], the resulting variability in the individual probabilistic representation might be substantial in detail. The second limitation is that the voice leadings in the present stimuli across conditions were not identical and the melody contour plays a major role in music perception. Although melodies alone can elicit ERAN-like response [50], the different melody contours in the present study are unlikely to have contributed to the EANm responses significantly. A recent study showed that irregular melodies elicited a negative ERP component earlier than irregular chords with the identical melody contours as the top voices and such a component diminished around 180 ms after stimulus onset [56]. However, it would be fair to note that the possible effect of melody contours may have affected the observed responses since the melodies covaried with chord functions in this study. Thus the present results should be carefully interpreted. Finally, though we have focused only on the final chord transition with the last two chord functions, further considerations on the previous transitions along the first four chords are needed to parameterize musical stimuli more comprehensively as in computational models [57].

In conclusion, the present study found a negatively correlating effect of conditional probability of chord progression on brain response. In addition, this relationship was found to be facilitated by musical training. As the neural response correlated with the behavioral performance, these findings suggest a physiological reflection of the probabilistic representation of the musical syntactic structures.
